# Under-Drilling versus Hybrid Osseodensification Technique: Differences in Implant Primary Stability and Bone Density of the Implant Bed Walls

**DOI:** 10.3390/ma13020390

**Published:** 2020-01-15

**Authors:** Rafael Delgado-Ruiz, Joshua Gold, Tanya Somohano Marquez, Georgios Romanos

**Affiliations:** 1 Department of Prosthodontics and Digital Technology School of Dental Medicine, Stony Brook University, Stony Brook, NY 11794-8712, USA; jdgold18@gmail.com (J.G.); Tanya.SomohanoMarquez@stonybrookmedicine.edu (T.S.M.); 2 Department of Periodontology, School of Dental Medicine, Stony Brook University, Stony Brook, NY 11794-8712, USA; georgios.romanos@stonybrookmedicine.edu

**Keywords:** osseodensification, under-drilling, primary stability, bone density

## Abstract

The goal of this study was to evaluate the effects of two implant bed preparation techniques on the implant primary stability (IPS) and the bone density of the implant site. We completed 40 implant bed osteotomies in pig ribs using two techniques: osseodensification (OD) plus under-drilling (UD) with universal osseodensification drills (Test A), and under-drilling alone with drills of the same implant system (Test B). Implants with a 4.1 mm diameter and 10 mm length were inserted, and the IPS was evaluated with three methods: (insertion torque (IT), periotest (PTV), and resonance frequency analysis (RFA). The bone density was evaluated using micro-computed tomography. ANOVA and Tukey’s post-hoc test were used for comparison of the IPS values, and Kruskal–Wallis was used to evaluate the bone density. Statistical significance was set at *p* < 0.05. The tested B technique (UD) achieved a higher IPS compared to the Test A technique (OD + UD) for all the evaluation methods (*p* < 0.05). Bone density was higher at the apical and middle region in Test A compared to Test B and control sites (*p* < 0.05). We concluded that although the bone density increased with the hybrid OD technique with universal drills, implant beds prepared with UD using drills with geometry similar to that of the implant are more efficient at increasing IPS values.

## 1. Background

Dental implant stability has a significant impact on the success of a dental implant [1]. This stability can be achieved through two methods: the mechanical stability established between the implant and the surrounding bone at the time of surgery or primary (initial) mechanical implant stability (IPS) [2], and the biological stability achieved through the osseointegration process and bone remodeling during the functional life of the implant (secondary implant stability) [3,4].

Higher IPS values are essential for immediate loading, since premature or excessive loads can produce micromotion [5]. Micromotion above 100 microns can lead to bone resorption and the formation of a non-mineralized implant–bone interface (fibrous encapsulation) [6,7]. The presence of osseointegration is clinically identified via the asymptomatic fixation of the implant to the surrounding bone, and histologically via direct contact between the bone and the implant without the interference of the soft tissue [8,9,10]. Various factors contribute to higher IPS values, such as bone quality and quantity [11], implant design [12,13], and surgical technique [14,15]. Also, systemic factors, such as age, metabolic diseases (diabetes, osteoporosis), and smoking can negatively influence secondary implant stability [16,17,18].

Considering bone quality and density, Lekholm and Zarb classified the human posterior maxilla as a bone type III or IV (characterized by a thin cortical plate and large trabecular spacing) [19]. In such bone quality, obtaining enough IPS for immediate loading procedures (>35 Ncm) is challenging due to inadequate bone volume around the implant’s surface [20,21], and early implant failure rates can increase [22].

The conventional implant bed preparation involves the use of drills with increasing diameters; the final drill diameter is comparable to the diameter of the implant being placed [23]. Modifications to the conventional implant bed preparation involve variations in the final drill diameter and sequencing or different drill designs applied to the surgical technique to increase the IPS [24,25,26,27,28,29,30,31]. Among these techniques are osseodensification (OD) [24,25,26,27,28] and, to undersize the osteotomy, the under-drilling (UD) technique [29,30,31].

OD is a technique that involves bone preservation and condensation [24]. In this technique, universal drills with a special tapered design operate in a clockwise or counterclockwise direction, thus enabling bone cutting or bone expansion and densification, respectively [25,26]. This technique comparable to the osteotome technique because it preserves and condenses the bone when the drills are used in a counterclockwise rotation, and thus can increase the IPS [27,28].

With the UD technique, an osteotomy is created that is smaller in diameter than that of the implant, to be inserted during the preparation [29]. As a result, the contact between the walls of the osteotomy and the implant surface increases, leading to a press fit situation that also enhances the IPS [29,31].

Neither excess of compression nor lack of stability are beneficial for osseointegration [32,33,34]. Studies have shown that when an implant is inserted with higher levels of friction in an excessively undersized osteotomy, microfracture and compression necrosis of the bone can occur, resulting in delayed healing [32]. When an implant is inserted with poor initial stability, micromovement and delayed osseointegration can lead to potential early failure in the case of uncontrolled loads [33]. Therefore, a proper equilibrium should exist between the amount of compression needed and a lack of physical contact with the surrounding bone [34].

In cases with very low-density bone, combining techniques that increase the IPS could be beneficial, thereby increasing initial bone-to-implant contact (BIC) without negatively affecting bone healing. Few reports have been published about combined drilling techniques [35], and no references could be found for combining drilling techniques with OD drills.

Consequently, the goal of this study was to compare the IPS and the trabecular bone density of the implant bed wall sites, prepared using two different techniques. The first technique was a new hybrid technique incorporating UD with OD. The second technique was UD alone. The null hypothesis is that no difference exists in IPS and trabecular bone density between the hybrid technique (UD and OD) compared to the UD technique.

## 2. Materials and Methods

### 2.1. Sample Distribution

We completed 40 implant bed preparations in 20 pig ribs. Twenty implant beds were completed with the UD + OD hybrid technique (Test A) and 20 using the UD technique alone (Test B).

The sample size (*N* = 40; *n* = 20 samples/group) was calculated using the following formula

*n*_1_ = (*σ*^2^_1_ + *σ*^2^_2_/*K*)(*z*1 − *α*/2 + *z*_1_ − *β*)^2^ Δ^2^ *k* = 1 (1)

where Δ is absolute difference between means*; σ*_1_ and *σ*_2_ are the variance of Mean 1 and 2, respectively; *n* is the sample sizes of the groups; *α* is the probability of type I error (0.05)*; β* is probability of type II error (0.2)*; z* is the critical Z-value; and *k* is ratio of sample size of Group 2 to Group 1.

### 2.2. Experimental Procedures

**Figure 1:**
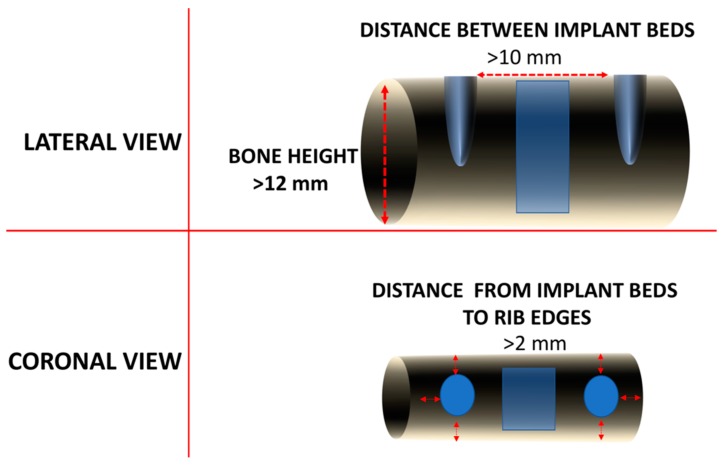
Scheme showing the location of the future implant bed osteotomies (grey colored). The lateral view shows that a minimum height of 12 mm was required to ensure space for the implant insertion (red dotted line). The distance between implants was more than 10 mm. The blue rectangle is the control area for the evaluation of bone density. The coronal view allows to observe that, in addition to the 4.1 mm space for the future implant insertion, at least 2 mm were present to avoid bone fractures. The blue circles are the future implants and the blue square is the control area for the evaluation of bone density.

Fresh pig ribs for this ex-vivo experiment were obtained from the butcher, and immediately processed for the experimental procedures as follows: decontamination with antiseptic, removal of the soft tissues with scalpel and periosteal instruments to expose the underlying bone, fixation and immobilization of the ribs in a vice grip device to the bench top, and then orientation with the posterior rib edge facing upward (convex side) and the anterior rib edge facing downward (concave side). Upon exposure of bone, the sites of the ribs with a width >9 mm and a height >12 mm were marked, maintaining a distance of at least 10 mm between marks, and providing at least 2 mm of bone from each edge to prevent fracture of the ribs during drilling (Figure 1).

#### 2.2.1. Implant Bed Preparation

Versah universal tapered drills (VT, Versah LLC, Jackson, MI, USA), with a 3.5 mm major diameter and 10 mm length, were used for the preparation of the Test A group implant beds. Zimmer tapered drills (Driva drill, Zimmer Biomet, Palm Beach Gardens, FL, USA), with 3.4 mm major diameter and 10 mm length, were used for the preparation of Test B group implant beds. The operator’s drilling was calibrated after repeating the drilling procedures 10 times per group when the intraclass coefficient (ICC) reached a value >0.8. A second operator recorded the primary stability data.

Each rib received two implant bed preparations, each one completed with a different drilling technique (hybrid drilling protocol and under-drilling technique).

The hybrid drilling protocol for the UD + OD technique (Test A) was completed using a pilot drill of 1.5 mm diameter followed by tapered universal drills with incremental diameters of 2.0 mm (ref. VT1525) and 3.0 mm (ref. VT2535) with counterclockwise rotation, with the exception of the pilot drill. The major diameter of the final drill was 3.5 mm and the minor diameter was 2.5 mm.

The drilling protocol for the UD technique (Test B) involved the use of tapered drills from the same implant system. Starting with a pilot drill, incremental diameters of 2.3 and 2.8 mm, ending with 3.4 mm diameter drills, were all used with clockwise rotation. The major diameter of the 3.4 mm drill was 3.4 mm and the minor diameter was 3.1 mm.

Drilling speeds were standardized as recommended by the manufacturers, and irrigation was applied for both implant bed preparation methods.

#### 2.2.2. Implants

We used 40 Zimmer tapered implants (Screw-vent^®,^ Zimmer Biomet, Palm Beach Gardens, FL, USA), with an internal hexagonal connection, diameter of 4.1 mm, and length of 10 mm, providing an implant with a wider diameter that that of the implant bed preparations completed for both groups.

#### 2.2.3. Implant Insertion

After the implant beds were completed, titanium dental implants were inserted using implant motor (FRIOS^®^, W&H Dental Werk GmbH, Buermoos, Austria) until the whole implant platform was level with the cortical bone.

#### 2.2.4. IPS Evaluation 

The IPS was evaluated using three methods: insertion torque, periotest, and resonance frequency analysis.

The insertion torque (IT) was recorded with the implant motor (FRIOS^®^, W&H Dental Werk GmbH, Buermoos, Austria). The IT required to insert the implant platform to the level of the cortical bone (flush) was registered in Ncm.

After the implant insertion, the Periotest device (Periotest^®^, Medizintechnik Gulden, Modatautal, Germany) was used for the evaluation of the periotest value (PTV) and the Osstell device (Osstell^®^, Brownstown, MD, USA) was used to measure the implant stability quotient (ISQ). The PTV was obtained by positioning the tip of the instrument perpendicular to the axis of the implant transfer. The average of three consecutive measurements was calculated per implant.

The ISQ values were obtained with the Osstell device by inserting a smart peg (Type 32 # 100440 Osstell^®^, Brownstown, MD, USA) into each implant after removing the implant transfer. The Osstell wand was placed at three random locations around the smart peg, and the mean of the three ISQ values obtained was calculated.

#### 2.2.5. Evaluation of Bone Density

After the evaluation of the implant’s primary stability, the implants were removed by reverse torque (to eliminate the metallic halation produced by the titanium implant that could hinder the evaluation of the bone’s microarchitecture) [36], and 10 of the implant beds were randomly selected per test group. An additional non-prepared area was selected on the same ribs to be used as a control for evaluation of native bone density. The area of evaluation was located in the central portion limited by both implant beds. The bone density was obtained in a micro-positron emission tomography/computed tomography (CT) scanner (Siemens Inveon^®^, Siemens Medical Solutions, Knoxville, TN, USA).

The settings for each scan were: voltage of 65 V, current of 190 μA, with a 360-degree rotation, and rotational step of two degrees per rotation, exposure time of 2500 ms, and a pixel size 21.07 µm. The thickness of each slice was standardized to 10 µm for a total of 850–1200 slides per sample. The scanning time per sample ranged between 30 and 45 min. The samples were positioned in the supporting area of the scanner, oriented with their longitudinal axis parallel to the scanning dock. After confirmation of orientation, the samples were secured, and a preliminary quick scan was completed for confirmation of the scanning time, slice thickness, and calibration.

The description of the trabecular spacing characteristics was obtained using Image J software (Image J, U.S. National Institutes of Health, Bethesda, MD, USA). Segments including the implant beds and the surrounding bone limiting the implant beds with a squared area of 15 × 12 mm were isolated. The areas were transformed to 16 bit images, and thresholding was completed to allow use of the interconnectivity plugging for the evaluation of the trabecular spacing characteristics.

The bone density was compared between the three groups: Test A (area prepared with UD + OD technique), Test B (area prepared with UD technique), and Control (unprepared area located between the test sites).

For the analysis of the bone density around the implant beds, we loaded Digital Imaging and Communications in Medicine (DICOM) data from each sample in the medical DICOM viewer open software available at Horosproject.org (Horosproject®, Nimble Co LLC, Annapolis, MD, USA)

Afterward, bone density analysis was completed using Image J software (Image J, U.S. National Institutes of Health, Bethesda, MD, USA).

**Figure 2:**
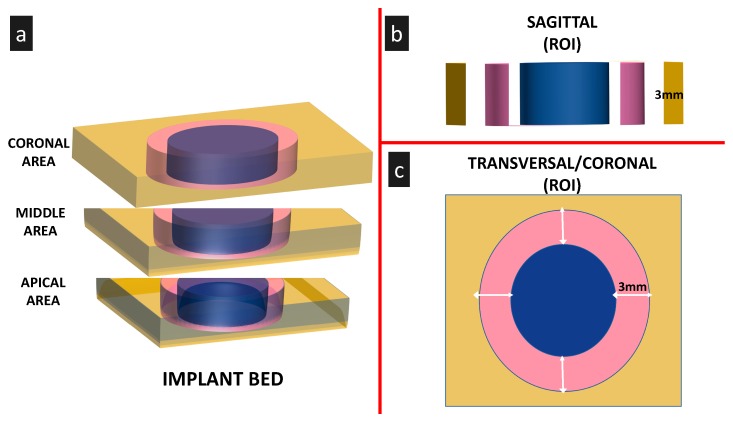
Scheme demonstrating the regions of interest for the evaluation of the bone density. **a**. Three segments were identified in the axial view (coronal, middle and apical thirds). **b**. In the sagittal projection, the bone density was measured in an area of 3 × 3 mm vertical and transversal. **c**. At the coronal projection, a circular area of 3 mm lining the implant bed walls was selected (pink area) and the bone density was measured at 36 random spots within the within the areas of interest (ROIs). Values were expressed in Hounsfield units.

The plug-in Stractec pQCT V.18 (Stratec Medizintechnik GmbH, Pforzheim, Germany) for Image J was used following the protocol described by Rantalainen et al. [37]. The regional bone mineral density, based on Hounsfield units [37], was measured in the sagittal and coronal sections at three different areas (coronal, middle, and apical regions) in a 3 mm zone lining the implant bed walls (region of interest) (Figure 2). 

The areas were isolated by manual tracing of the outer and inner edges of the regions of interest (ROI). As recommended by Rantalainen et al. [37], the most external and the most internal pixels limiting the area were cleaned to eliminate volume defects. The isolated areas were selected and the bone density was calculated from thirty-six random measurements per area. Mean and standard deviations of the measurements obtained per area per group were registered.

### 2.3. Statistical Analysis

The Shapiro–Wilk test was used to determine the normality of the samples. Analysis of variance (ANOVA) and Tukey’s post-hoc test were used for statistical comparisons of IT, periotest values, and ISQ. To evaluate the bone density non-parametric data, the Kruskal–Wallis test was used because the sample did not follow a Gaussian distribution. Mean values ± standard deviations and median values with upper and lower quartiles were compiled. The statistical significance was set as *p* < 0.05.

## 3. Results

### 3.1. Implant Primary Stability

The Test B preparation had demonstrated significantly higher IPS values with IT (IT: 32.61 ± 2.0 Ncm) compared to the Test A preparation method (IT: 25.24 ± 2.5 Ncm). The PTV indicated that UD with the drills of the same system (Test B) provided significantly higher stability than UD + OD with Versah drills (Test A). The ISQ had similar results: the UD protocol showed significantly higher ISQ values (ISQ: 78.55 ± 0.10) compared to UD + OD (ISQ: 63.05 ± 0.40). (Table 1).

### 3.2. Micro-CT Evaluation

**Figure 3:**
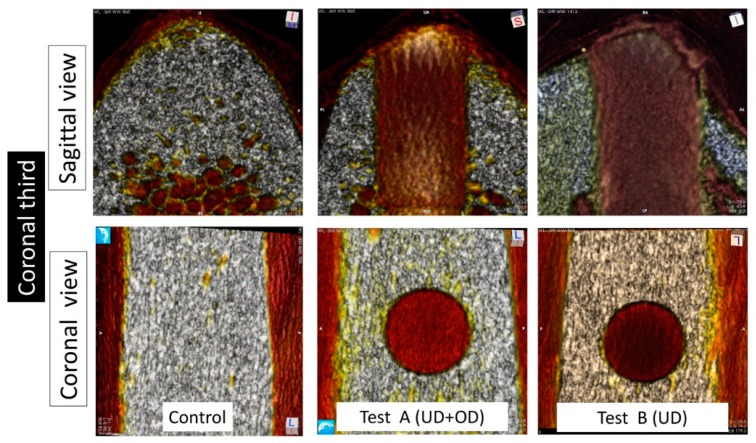
Micro-computed tomography (CT) image obtained from the coronal third at sagittal and coronal projections. In the sagittal projection, a cortical thickness of around 3 mm was observed in all the groups. The BD was similar in the control and test groups.

The analysis of the images at the sagittal view showed a thick cortical layer around 3 mm thick in all groups (Figure 3). The trabecular area in the middle and apical regions differed between groups. In the control group, the trabecular pattern was comparable to that of Type III bone [19]. In both Test A and Test B groups, the walls created by the implant bed preparation presented a thickening of the trabeculae of 0.5 to 1 mm at the middle and apical thirds. In the coronal cortical region, these differences were barely noticeable among the groups.

**Figure 4:**
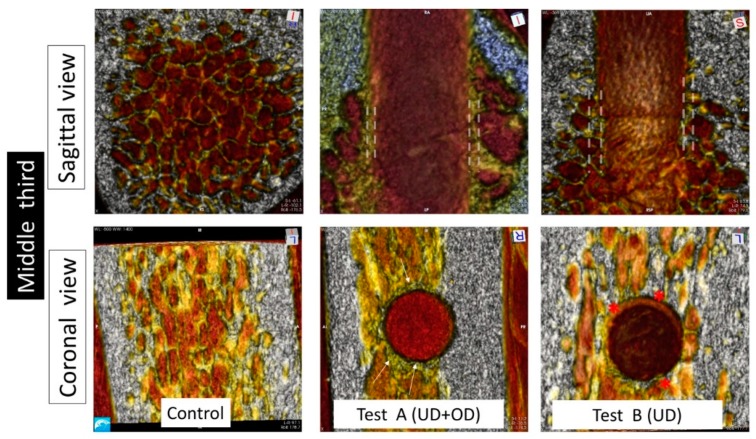
Micro-CT image obtained from the middle third at sagittal and coronal projections. At the sagittal projection, the Control group showed a trabecular pattern coincident with Type III bone. The Test A group showed the space of the prepared implant bed surrounded by a thick wall of bone of around 1 mm thick (white dotted lines). The Test B also showed an empty space in the implant bed surrounded by a wall of around 0.5 mm thickness (dotted white lines). At coronal projections, the Control group showed the same trabecular pattern described at the sagittal projection. The Test A group showed a defined circumferential dense bone wall completely surrounding the empty implant bed space (white arrows). The Test B group showed localized thickening of the implant bed walls interrupted by trabecular bone (red dots).

**Figure 5:**
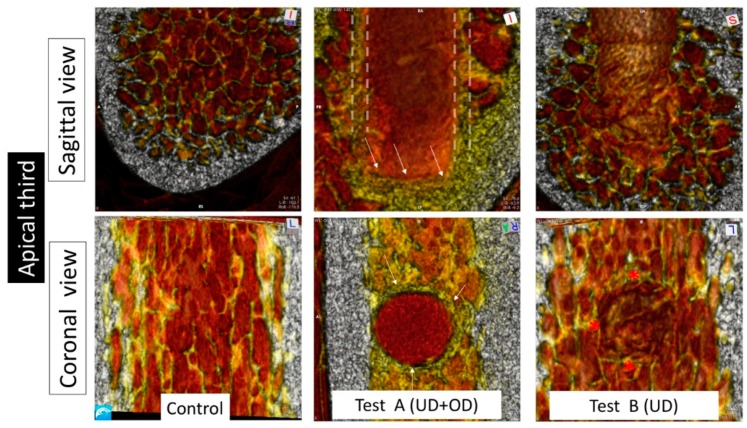
Micro-CT image obtained from the apical third at sagittal and coronal projections. In the sagittal projection, the control group showed a trabecular pattern coincident with Type III bone and the cortical bone at the basal region. The Test A group showed an empty space in the implant bed with highly dense bone at the apex (white arrows), and dense bone forming the implant bed walls (white dotted lines). The Test B group showed an empty space in the implant bed and broken trabecular bone forming the implant bed walls. From the coronal view, the Test A group showed dense bone walls (thicker than in the middle region), identified by white arrows. The Test B group showed localized thickening that was denser than the Control group.

The comparison of the images of the transversal view of the coronal regions showed minimal differences for all groups (Figure 3, Figure 4 and Figure 5). In the middle and apical areas, we observed bone densification/thickening of the implant bed walls coincident with the findings of the sagittal view of around 0.5 mm for the Test B and around 1 mm for the Test A groups (Figure 3, Figure 4 and Figure 5).

Test A and Test B groups displayed higher bone densities compared to the controls at the middle and apical regions in sagittal and transversal evaluations. In comparison with Test B, Test A showed superior density in the middle area of the implant bed walls, and Test A showed the highest apical density amongst all groups (Table 2).

## 4. Discussion

In this experimental study, we tested two methods used for implant bed preparation that enhances the IPS. For the implant bed preparation with the hybrid OD + UD technique, we used universal tapered osseodensification burs with narrow major and minor diameters (3.5 and 2.8 mm) compared to that of the implant diameter (4.1 mm). For the UD technique, we used implant system burs with a geometry similar to that of the implant, but with narrower major and minor diameters (3.4 and 3.1 mm).

In this experiment, pig ribs were chosen, among other bone models, for the evaluation of the implant stability and the trabecular changes because the pig ribs possess similar collagen content, and density, trabecular pattern, and cortical and cancellous bone ratio similar to that of the human bone with Type II and III density [38,39].

The IT, PTV, and ISQ results indicated that using UD with system drills produced a significantly higher IPS compared to UD + OD with universal osseodensification drills. The findings can be explained by differences in the diameter of the osseodensification drills and the system drills that, for the OD + UD technique, produced 0.6 mm of under-sizing (14.63%) and, for the UD technique, 0.7 mm of under-sizing (17.07%) at the coronal area. Also, the tapered drills used for the preparation of the implant bed with the UD technique have a geometry that matches the geometry of the implant being inserted. Thus, the initial bone-to-implant contact increases along the entire implant surface. In contrast, the universal osseodensification drills, although tapered, do not match the implant geometry, resulting in less initial bone-to-implant contact and empty spaces in certain areas around the implant body.

Our results agree with those reported by Shalabi et al. [40], who evaluated the influence of the preparation method on implant stability. Their findings showed that when the preparations were undersized (last drill of 4.0 mm diameter and implant body 4.6 mm), the IPS increased compared with other implant bed preparation methods. González-Martín et al. [41], in a study in humans, found that immediate implants with a slightly wider apical region (3.5 mm) than the implant bed preparation (2.5 mm), undersized by 28.5%, had excellent IPS values and did not undergo adverse changes in bone quality, as evaluated by cone beam-CT at six months.

Marin et al. [42] compared different undersized osteotomies (3.0, 3.25, and 3.5 mm) for the insertion of 3.75 mm diameter implants. Their results showed that the implant IT values increased in narrower osteotomies without negatively affecting the osseointegration process observed in posterior histological analysis. Pantani et al. [43] showed that implant bed preparations with drills 0.2 mm narrower than the inserted implants did not negatively affect the bone to implant contact after four months.

The osseodensification technique, compared with conventional drilling protocols, increases the IPS [44,45,46]. Lahens et al. showed in a sheep model that the osseodensification technique increased the insertion torque of conical and paralleled-wall implants [44]. Oliveira et al. evaluated the effects of the osseodensification technique on IPS and the progression of the osseointegration in the iliac crest of sheep. Their results showed that the osseodensification increased the insertion torque by almost 60% compared with the conventional drilling protocol, which favored early osseointegration [45]. Tian et al. [46] compared osseodensification with the osteotome technique to increase primary stability. Their results showed increased insertion torque values for implants inserted in beds prepared with osseodensification, compared to implants inserted in beds prepared with the osteotome technique.

The literature shows that IPS is a static mechanical parameter that is mostly reflected and measured by the implant IT. There is not a minimum of IT value required for submerged implants. However, for single implants requiring immediate loading, an IT value range between 30 to 35 Ncm has been recommended to reduce implant micromovement [47]. The periotest allows initial and repeated measurements of the implant’s stability and, when compared to the IT parameter, is significantly correlated with predicting implant stability but with considerable variability [46]. Negative PTV values indicate the implant’s stability for potential immediate load and positive higher values indicate implant micromovement and potential contraindication for implant loading [48]. The resonance frequency analysis has demonstrated reliability in in vitro studies, [49] and values over >60 ISQ allow immediate loading. However, variations in the tightening force of the transducer on the implant can increase the variability of the stability measurements [50].

Given the potential inherent errors in the individual techniques, we selected a multimodal approach including three different methods of evaluation (insertion torque, periotest, and resonance frequency analysis). All three methods consistently demonstrated increased primary stability with the Test B technique (under-drilling) compared to the Test A technique (osseodensification + under-drilling).

The integrated device that we used for the evaluation of bone density is a hybrid Positron Emission Tomography (PET) scan/micro CT scan that allows either PET or micro-CT studies of a single integrated gantry. This multimodality system fully integrates each modality into a common data acquisition system for automatic transition between modes and seamless coordination of CT and PET data acquisition. For this study we used just the micro-CT option.

Micro-CT was used to understand the effects of both implant bed preparation techniques on bone microarchitecture and bone density. We observed that the bone microarchitecture changes displayed two characteristics: the thickening of the implant bed walls and the reduction in the trabecular spacing around the implant bed walls. These changes were profound in the middle and apical regions in both groups but the highest density was observed in the apical region of the OD + UD group.

These phenomena can be explained for both groups as follows. For the under-drilling + osseodensification group, the universal osseodensification tapered drill has a cutting tip that cuts the cortical bone and perforates the trabecular bone during its displacement. Displacement of the drill in the apical direction produces compaction and additional condensation of the bone fragments fractured by the drill tip toward the apical area (effect of the drill tip). Simultaneously, the lateral walls of the drill compress and compact the bone structure laterally (effect on the drill walls). Afterwards, an implant with a wider body produces supplementary compaction due to its bigger dimensions (effect of the implant insertion). In other words, the characteristic bone density pattern observed in the micro-CT evaluations of the under drilling + osseodensification technique was produced by the drill design (tip and walls), drill rotation scheme, drill displacement, and implant insertion.

For the under-drilling alone group, the drill geometry matches the implant geometry but not its dimensions; the drill cuts and removes bone but leaves a smaller implant bed with a smaller diameter of that of the implant. The under-drilling does not increase the bone density, but the implant body pushes, fractures, and compresses the bone trabecula, creating walls with increased density (effect of the implant insertion) in the middle and apical regions. In other words, the characteristic bone density pattern was produced solely by the insertion of an implant with larger dimensions and a similar geometry to that of the drills used.

The former concept agrees with an experimental study by Slete et al. [51], who compared the structure of the osteotomy prepared by conventional drilling, Summers osteotomes, and osseodensification using histomorphometric analysis. The authors found higher bone-to-implant contact for osseodensification and Summers techniques compared to conventional drilling, demonstrating that the surgical technique changes the original implant bed characteristics [51].

The osseodensification method increases the bone density but is not superior to under-drilling, or potentially to other methods related to increasing the primary stability. This finding is in agreement with a systematic review by Tretto et al. [52], who evaluated the influence of the instrument used for the implant bed preparation in the bone–implant interface characteristics. Their systematic review identified standard regular drills, osteotomes, piezoelectric surgery, surgery with an Er:YAG laser, and osseodensification drills. Their results showed that depending on the variable evaluated, some instrumentation methods were superior than others [52].

Finally, the osseodensification drill, rotating in a clockwise or counterclockwise direction, also produces bone fractures and not just bone compaction, evidenced by histological findings of bone chips adjacent to the implant’s body inserted in sheep’s ilium [53]. These bone chips can contribute to increased primary stability through an increase in the frictional contacts.

The limitations of the present work are the small number of samples used for the micro-CT evaluations and the lack of a control group for the evaluation of the IPS values. However, the inclusion of a control area in the same rib, evaluated by micro-CT, allowed the comparison of the changes in bone density induced by both techniques in each rib. Also, both techniques being applied in the same ribs excludes site variability between ribs.

Finally, our experiment lacked support from additional histological analysis, which could define the bone-to-implant contact (BIC) achieved using each technique. However, the micro-CT technique has been described as comparable to histology, with the advantage of a 360° visualization and specificity for the evaluation of the bone’s microarchitecture [54,55].

Notably, although sufficient primary stability is required for immediate loading protocols, it is not a pre-requisite for the implant osseointegration, as was demonstrated by implants that were submerged or properly stabilized, that were able to osseointegrate in cases with low primary stability [56].

Two final points for future in vivo study are the potential effects of osseodensification on the trabecular spacing, which can produce occlusion/reduction of the vascular supply and an excess of compression produced by the under-drilling technique, that could result in bone necrosis.

The strength of the present work is the strict operator calibration and the use of a second operator for obtaining the IPS and bone density measurements (thus, reducing bias). Furthermore, the IPS was evaluated with three methods that consistently showed agreement in the evaluation of the IPS. This is the first work that combined osseodensification with other proven techniques for an increase in IPS. The clinical significance of the present work is that under-drilling with drills of the same implant drill system is more efficient than using the hybrid osseodensification technique when attempting to increase IPS.

The universal osseodensification drills (as per the manufacturer) can be used with many implant systems. However, based on our results, their configuration should be similar to the implant geometry to improve the primary stability and extend the benefits of the technique to a broader range of implant systems.

## 5. Conclusions

The null hypothesis was rejected. We found differences in implant primary stability and bed walls’ density when UD was compared to OD + UD.

- Preparing the implant bed using the UD technique with drills with a similar geometry to the implant being inserted provides superior implant primary stability (IPS) than using universal osseodensification drills with UD;
- Preparing the implant bed using the OD technique with universal drills changes the bone microarchitecture and increases the bone density in the middle and apical areas of the implant bed;
- Implant bed preparation with the UD technique using drills of the same system does not change the bone microarchitecture or the bone density;
- The insertion of an implant with a wider diameter and the same geometry as the drill changes the bone microarchitecture and increases the bone density in the middle and apical regions of the implant bed.

## Figures and Tables

**Table 1:** Implant primary stability evaluation. Significantly higher stability was observed for implants inserted in Test B (under-drilling (UD) group) compared to Test A (UD + osseodensification (OD) group) evaluated with periotest and implant stability quotients. The symbol "*" represents higher values and statistical significance.

**IT** **(Insertion Torque) (Ncm)**	**Sample Size**	**Mean**	**Median**	**Sum of Ranks**
**Test A**	20	25.246	25.717	480
**UD + OD (Versah)**	-	-	-	-
**Test B**	20	32.611 *	33.042	652
**UD (Zimmer)**	-	-	-	-
**H**	10.250		H (corrected)	
**Degrees of Freedom (DoF)**	1		N	
***p*-value**	0.0038 *			
**PTV** **(Periotest value)**	**Sample Size**	**Mean**	**Median**	**Sum of Ranks**
**Test A** **UD + OD (Versah)**	20	−2.4025	−2.675	516
**Test B** **UD (Zimmer)**	20	−4.69833 *	−4.65	304
**H**	8.22146		H (corrected)	
**DoF**	1		N	
***p*-value**	0.00414 *			
**ISQ** **(Implant Stability Quotient)**	**Sample Size**	**Mean**	**Median**	**Sum of Ranks**
**Test A** **UD + OD (Versah)**	20	63.05	68.5	280.5
**Test B** **UD (Zimmer)**	20	78.55 *	82	539.5
**H**	12.27091		H (corrected)	
**DoF**	1		N	
***p*-value**	0.0046 *			

**Table 2:** Bone density at transversal and sagittal sections. The Test A group showed significantly higher density in the apical and middle areas compared to Test B and Control groups. the Test B group showed a higher density in the middle third compared to group C in the transversal section, but not in the sagittal area. Test B, in the apical section, showed higher bone density compared to the Control group. Superscript letters identify groups and their comparisons, the symbol "*" represents higher values and statistical significance.

**Implant Bed Walls** **Transversal Density Measurements**	**Test A^a^** **Mean ± SD** **(*n* = 10)**	**Test B^b^** **Mean ± SD** **(*n* = 10)**	**Control^c^** **Mean ± SD** **(*n* = 10)**	***p*-Value**
**Coronal**	221 ± 8	214 ± 7	208 ± 6	(a vs. b *p* = 0.052)
				(a vs. c *p* = 0.051)
				(b vs. c *p* = 0.06)
**Middle**	218 ± 10^b,c^	186 ± 11 ^c^	160 ± 12	(a vs. b *p* = 0.042 *)
				(a vs. c *p* = 0.024 *)
				(b vs. c *p* = 0.032 *)
**Apical**	232 ± 9^b,c^	215 ± 8 ^c^	189 ± 10	(a vs. b *p* = 0.049 *)
				(a vs. c *p* = 0.033 *)
				(b vs. c *p* = 0.047 *)
**Implant Bed Walls** **Sagittal Bone Density Measurements**	**Test A^a^** **Mean ± SD** **(*n* = 10)**	**Test B^b^** **Mean ± SD** **(*n* = 10)**	**Control^c^** **Mean ± SD** **(*n* = 10)**	***p*-Value**
**Coronal**	231 ± 5	229 ± 3	226 ± 4	(a vs. b *p* = 0.058)
				(a vs. c *p* = 0.06)
				(b vs. c *p* = 0.063)
**Middle**	204 ± 9^b,c^	194 ± 1	186 ± 3	(a vs. b *p* = 0.046 *)
				(a vs. c *p* = 0.036 *)
				(b vs. c *p* = 0.05 *)
**Apical**	221 ± 3^b,c^	209 ± 2 ^c^	199 ± 5	(a vs. b *p* = 0.048 *)
				(a vs. c *p* = 0.031 *)
				(b vs. c *p* = 0.046 *)
